# J. Foster Flagg, D.D.S.

**Published:** 1903-12

**Authors:** 


### J. FOSTER FLAGG, D.D.S.

Died, after a lingering illness, November 25, 1903, at Swarthmore, Delaware County, Pa., Dr. J. Foster Flagg.

The silent reaper has been rapidly decimating the ranks of the men who have done so much to make dentistry as we have it in this the earlier years of the twentieth century. It is with no ordinary feeling that the writer is called upon to record the fact that another has passed into the realms of silence after a life of unusual activity and one that has left a profound impression on the minds of his colleagues during the period in which he labored.

The time has not yet arrived when Dr. Flagg's work can justly be considered. He was a man not trained to follow in paths trodden by other men. If the word original can be applied to any one, it truly belonged to him, and in the way he marked out for himself few were found to follow; yet no man that dentistry has produced has made the impression upon his calling, in this country, as Dr. Flagg has done.

He came into his profession when dentistry was struggling from the chaotic period antedating the earlier efforts of the first half of the nineteenth century, and he was necessarily infused with the energy that characterized that period. This, combined with an exhaustless fund of original ideas, prompted him at once to take a leading part in the work of his day. He early became a leader, and blazed the path for himself through the tangled maze of practice which was just beginning to reach out to broader fields of activity.

No man in dentistry has been subjected to more adverse criticism than was his experience to endure, and while we may not agree with him in all his conclusions, especially in his views upon the—so-called—"New Departure," it must be said of him that he bore all this with kindly feeling, sure that in time justice would be done and the dental profession would eventually come to realize that there was a large substratum of truth underlying his attacks upon the dictum formulated by the fathers, "That what was worth filling at all was worth filling with gold." He taught "that in the tooth that most needed saving, gold was the poorest material to use."

The writer reserves for a future number to discuss this matter more in detail than would be proper at this time. It must be said, however, that he lived to see this apparently iconoclastic idea measurably adopted by the dental profession, not that gold was abandoned, but many of the old and severer ideas held upon this subject fell into decadence, never, probably, to be revived as they were held forty years ago.

Those who knew Dr. Flagg intimately greatly admired his brilliant conversational powers and facility of expression upon the public rostrum, as well as his ever-genial character. In associated work he was an aggressive controversialist, but he never harbored an unkindly feeling towards his opponents. He seemed to recognize, as few others, that opinions were often based on mental temperament and were subject to change as more intelligent thought led to broader conceptions.

His work was not based on mere theory. He was a tireless investigator in his own field of work. Few men have done more to perfect the various filling-materials that have been classed as alloys and plastics, and it is only justice to his memory and his labors to say that no one has materially improved on his products in this direction.

While Dr. Flagg had long been retired from active practice and dental educational work, he still maintained a lively interest in dental subjects. His declining health prevented a personal activity, but no one more enjoyed the occasional visits of old friends, when he could talk over the exciting scenes of past contentions.

Dr. Flagg is almost the last of a race of men of which he and Dr. Taft, so recently deceased, are marked exemplars, and yet two men more unlike could hardly be found in professional circles. They both, however, lived to see marked advances made in the direction of their ideals.

The writer feels that, in parting with Dr. Flagg, a link has been dropped out of the chain of association, and yet its loss leads to a closer bond with those who still remain to carry on the work. When one has passed the limit usually given to man, it is idle to express vain repinings. Our friend and coworker has finished his labor with us to complete it more perfectly, it is hoped, in the eternal round of activities given to all who aspire to the highest.

